# Contextual weighting for Support Vector Machines in literature mining: an application to gene versus protein name disambiguation

**DOI:** 10.1186/1471-2105-6-157

**Published:** 2005-06-22

**Authors:** Tapio Pahikkala, Filip Ginter, Jorma Boberg, Jouni Järvinen, Tapio Salakoski

**Affiliations:** 1 Department of Information Technology, University of Turku and Turku Centre for Computer Science (TUCS), Lemminkäisenkatu 14 A, 20520 Turku, Finland

## Abstract

**Background:**

The ability to distinguish between genes and proteins is essential for understanding biological text. Support Vector Machines (SVMs) have been proven to be very efficient in general data mining tasks. We explore their capability for the gene versus protein name disambiguation task.

**Results:**

We incorporated into the conventional SVM a weighting scheme based on distances of context words from the word to be disambiguated. This weighting scheme increased the performance of SVMs by five percentage points giving performance better than 85% as measured by the area under ROC curve and outperformed the Weighted Additive Classifier, which also incorporates the weighting, and the Naive Bayes classifier.

**Conclusion:**

We show that the performance of SVMs can be improved by the proposed weighting scheme. Furthermore, our results suggest that in this study the increase of the classification performance due to the weighting is greater than that obtained by selecting the underlying classifier or the kernel part of the SVM.

## Background

The amount of scientific biomedical literature readable by computer programs is overwhelming. For example, PubMed [1] contains about 7.5 million article abstracts. Therefore automatic literature-mining methods can be exploited in order to retrieve relevant information (for recent thorough reviews of related work in Bio-NLP, see e.g. [2,3]). For example, several algorithms have been developed for extracting information about protein-protein interactions from the biomedical literature [4-10].

### The problem

In order to find the relations between biological or chemical entities, first the names of the entities have to be recognized in a reliable way. There has been a significant amount of effort to do that automatically [11-20]. A large standardized domain corpus helps to consolidate the research efforts. The GENIA corpus [21] has been commonly used in biomedical named entity recognition. The state-of-the-art systems have recently been compared, for example, in Kim et al. [22] using the GENIA corpus.

The task of named entity recognition can be divided in two subtasks, the identification of entities, that is, determining the boundaries of the named entities, and their classification into proper classes. The problem of finding the class the entity belongs to can be treated as a word sense disambiguation (WSD) task, which, on its own, is an essential part of natural language processing (see e.g. Manning and Schütze [23] for more information).

The entities in biomedical text are highly ambiguous. For example, it is common that a gene has the same name as the protein it codes for. In the following three sentences from the GENIA corpus, the occurrences of BZLF1 are a protein, a gene, and an RNA, respectively: (1) *Expression of either BZLF1 or BRLF1 triggers expression of*... (2) ... *DNA in lymphoblastoid cell lines induced by transfection with BZLF1*. and (3) ... *lysis of certain HLA B8*+ *LCL targets was associated with the abundance of BZLF1 transcripts*. Similar ambiguity is illustrated in the two sentences given by Hatzivassiloglou et al. [24]: *By UV cross-linking and immunoprecipitation, we show that SBP2 specifically binds selenoprotein mRNAs both in vitro and in vivo. The SBP2 clone used in this study generates a 3173 nt transcript (2541 nt of coding sequence plus a 632 nt 3' UTR truncated at the polyadenylation site)*. The occurrence of SBP2 is a protein in the first sentence, whereas the occurrence of SBP2 in the second sentence is a gene. In the same study, a domain corpus was annotated by three biology experts. The three experts unanimously agreed only in 78% of the cases, each name being classified as either a gene, protein or mRNA. This low rate of inter-annotator agreement suggests that the task is relatively difficult even for human experts, reflecting the inherent complexity of the domain. However, the study does not analyse more closely the reasons that lead to annotation disagreements.

In this paper, we consider the disambiguation of the sense "gene" or "protein" when the name is not disambiguated explicitly by the author with the word "gene" or "protein" (e.g. "SBP2 gene"). This task is important, because the release of the human genome and large scale functional genomics studies and methods have made it important to be able to find information from literature specifically for proteins and the corresponding genes. However, database searches provide a lot of hits among which the correct and important articles have to be sorted manually. Therefore, for example, in data mining related to proteomics the scientists could save much time if they could direct their search only to proteins.

Much of the ambiguity in biomedical text is caused by inconsistent or non-existent naming conventions. For example, there exist Drosophila gene names such as *ring *and *arc *that can be confused with their ordinary meanings. Manual analysis of a small set of abstracts returned by PubMed for the query *ring and drosophila *shows that the word *ring *appears in its gene/protein sense in about 30% of the cases, both capitalized and non-capitalized. Similarly, the word *arc *is ambiguous and appears in about 75% of the cases in its gene/protein sense, again both capitalized and non-capitalized. In both cases, only abstracts regarding Drosophila were considered, thus the two example words retain their ambiguity even in the sublanguage of articles concerning Drosophila. In contrast, some other gene/protein names, such as, *tax *do not retain their ambiguity in the sublanguage: all occurrences of *tax *in the GENIA corpus refer to its gene/protein sense. Another major source of ambiguity in scientific biomedical text are abbreviations, which are widely used and therefore are very important to be identified correctly in natural language processing applications [25].

There have already been applications of word sense disambiguation methods in the field of scientific biological text processing. Hatzivassiloglou et al. [24] disambiguated names of genes, proteins and RNAs using a Naive Bayes classifier. Previously we have developed a method named here Weighted Additive Classifier (WAC) and applied it to the problem of gene/protein name disambiguation [26]. Liu et al. [27] disambiguated abbreviations from Medline abstracts using a Naive Bayes classifier. Yu et al. [28] achieved better results for the same task using Support Vector Machines (SVMs) with the one sense per discourse hypothesis. Furthermore, a system developed by Podowski et al. [29] assigns gene names to their LocusLink IDs in previously unseen abstracts.

Lee and Ng [30] performed a comparison of several supervised learning algorithms for WSD tasks and in their study, SVMs were confirmed to have the best performance. SVMs have also been applied in biomedical WSD (see e.g. Yu et al. [28]) as well as in biomedical named entity recognition [20,31-35]. Furthermore, in the COLING-2004 JNLPBA shared task of Bio-Entity Recognition [22], five studies [36-40] used SVMs either alone or combined with other algorithms. In this paper, we apply SVMs and as baselines, we consider the Naive Bayes and WAC classifiers. The studies mentioned above use narrow context windows and focus mainly on studying different features such as orthographical, morphological, lexical, contextual, part-of-speech, head-noun, and name-alias features. We, in contrast, focus on context representations that use distance of the words from the ambiguous name in addition to the context words themselves. We evaluate the methods on the GENIA data set (see e.g. Collier et al. [21]), using the area under ROC curve (see e.g. [41]) as a performance measure.

### Support Vector Machines

SVMs can be used to classify multidimensional data into two classes. SVMs were introduced by Boser et al. [42]. Thorough presentations of SVMs are given by Burges [43] and Vapnik [44], for example, and in the Methods section we give a concise introduction to SVMs. In a binary classification task, the training set consists of data points which are labeled as positive or negative. In our case, the training data points are the contexts of the ambiguous names. The positive and negative labels denote genes and proteins, respectively. In order to improve linear separability, the data points are mapped from the input space to a new feature space before they are used for training or for classification. The mapping is done implicitly by a so-called kernel function, which computes the similarity of two data points in the feature space. The choice of an appropriate kernel function is a nontrivial problem, but there are certain standard kernel functions which are frequently used. Kernels and the SVM itself also have certain parameters which have to be adjusted in order to make the SVM classifier work in the best possible way.

### Contribution of this work

In short, we considered application of SVMs to the gene versus protein name disambiguation problem in abstracts of biomedical articles. While other studies focus mainly on studying different features, our work primarily considers context representations. We resolve the ambiguous names using their context which spans up to the whole abstract, in contrast to other previous applications of SVMs which typically use narrow context windows. To improve the performance of conventional SVMs and accommodate the wide context span, we adopted a weighting scheme introduced by Ginter et al. [26] that exploits the information about the distances of the words from the name to be disambiguated, and adjusted the scheme for the SVM classifier. We carefully searched for the best parameter values of SVMs and kernel functions using grid optimization as suggested by Hsu et al. [45], and we also performed a similar search for the parameters of the proposed weighting scheme. Finally, we measured the performance of both conventional and weighted SVMs together with two baseline methods, and showed that the performance improvement was statistically significant.

## Results and discussion

We experimented with the protein versus gene name disambiguation problem using conventional SVMs with linear, Gaussian, as well as second and third degree polynomial kernels. Also, we tested SVMs using different kernels augmented with the proposed weighting scheme. As additional baseline classifiers, we used the Weighted Additive Classifier, which also uses contextual weighting, and the Naive Bayes classifier. These methods and their parameters to which we refer in this section are described in detail in the Methods section.

In the following, we first discuss the weighting scheme and the reasons why its use is beneficial. Then, we present how the data was generated and preprocessed. Finally, we present the performance measure used in the experiments, describe the experimental setting and the results of the parameter estimation and the final validation.

### Contextual weighting

The training data points are vectors of word frequencies in the context in which the names to be disambiguated were found. The basic SVMs with any kernel use only the word frequencies and do not take into consideration the distances of the words with respect to the position of the name to be disambiguated. However, the distance information seems intuitively to be important, and therefore we apply a weighting scheme that incorporates this information into the context representation used by SVMs. The weighting scheme models the distances of the words from the ambiguous name, while the information whether the words are before or after the ambiguous name is not considered. The weight of a context word at the distance *d *is given by *d*^-*λ *^+ *β*, where the parameters *λ *and *β *are used to control the effect of the distances of the words from the name to be disambiguated. A more detailed explanation of the weighting scheme is presented in the Methods section.

**Figure 1:**
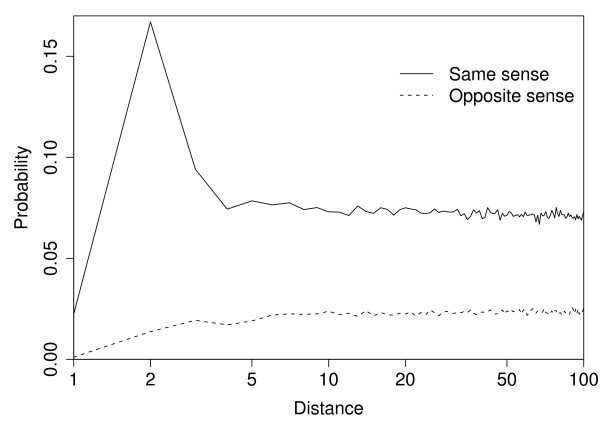
**Conditional probabilities of having other instances of ambiguous names in the context of the name to be disambiguated. **The X-axis denotes the distance in words from the name to be disambiguated and Y-axis is the probability. The solid line denotes the probability that if there is a word in the context at the given distance, the word would be another ambiguous name having the same sense with the name to be disambiguated and the dashed line denotes the corresponding probability for the opposite sense.

We now discuss some possible reasons for the weighting scheme achieving a statistically significant gain in classification performance. Yarowsky [46] argues that the effect of context words is strongest for immediately adjacent words, and weakens with distance. This phenomenon is called the *one-sense-per-collocation *principle. Yarowsky also considers the *one-sense-per-discourse *principle, that is, all instances of an ambiguous word tend to have the same sense within one discourse unit, the article abstract in our case. In that case also distant words can help in disambiguation. One-sense-per-discourse is, however, presumed to be a weaker hypotheses, which should be overridden when the local evidence is strong. In order to study the tenability of these hypotheses in our data, we estimated the following conditional probabilities of the name to be disambiguated to have another instance of an ambiguous name in its context. For each distance from the name to be disambiguated, we estimated the conditional probability that there is a word in the context at that distance and the word is another ambiguous name with the same sense, as well as the conditional probability that the word is a name with the opposite sense. These probabilities are illustrated in Figure 1, where the solid line denotes the probability of an occurrence of a name with the same sense and the dashed line denotes the probability of the opposite sense. At close distances (*<*6), the probability of the same sense turned out to be high and decreasing with distance, whereas the probability of the other sense behaved in the opposite way. In the proposed method, the words in the area of influence of the one-sense-per-collocation principle, that is, the words at close distances, have more weight than the long distance words and these weights are controlled by the parameter *λ*. On the other hand, at long distances, the probabilities of the same and the opposite senses settled down to 0.08 and 0.02, respectively, indicating that mostly the one-sense-per-discourse principle holds. Therefore, when the close context is unable to make a strong decision, the information of the long distance words may be useful. This effect is controlled by the *β *parameter, which balances the influence of the one-sense-per-discourse principle compared to the one-sense-per-collocation principle. Note that one-sense-per-discourse does not have to hold strictly, because the information can be useful if there are on average more instances of the names with the same sense than with the opposite sense in the far context. Both near and far context words are important when deciding the sense of a name. For example, verbs like "activate" or "phosphorylate" are often found around protein names, whereas verbs like "express" or "transcribe" may be found around gene names. Similarly, head nouns, such as expression, are also highly indicative of the sense. These words may be located near to the name to be disambiguated, being strong indicators of its sense. As shown above, ambiguous names in the abstract are more likely to be of the same sense and therefore the words around the other ambiguous names are partly indicative about the sense of the name to be disambiguated. Since other ambiguous names can occur at any position of the abstract, it is beneficial to use long context. Descriptions of experimental conditions can indicate one sense common to all of the names in the abstract. For example, "yeast two-hybrid" indicates protein-protein interaction finding, while "microarray" relates to gene experiments. Further, the occurrence of a distant coreference, for example, between the full form of an ambiguous name and its abbreviation, in the context of the ambiguous name may provide distant words indicative of the correct sense.

The phenomena discussed above are highly data dependent. However, the proposed weighting scheme models them if the weighting scheme is equipped with the optimal values of the parameters *λ *and *β *found in the training phase.

### Data and its preprocessing

The data set considered in this paper is constructed as follows. We obtained the evaluation data set for the COLING-2004 JNLPBA shared task of Bio-Entity Recognition [22], which is derived from the GENIA corpus [21], a standard corpus for biomedical named entity recognition, by conflating the original 36 classes into five classes (DNA, RNA, protein, cell line and cell type) of which we only use two classes, namely, DNA and protein. The data set consists of 2000 hand-annotated abstracts and contains 30269 protein examples and 9533 DNA examples. Average number of words in the abstracts is 246.

Naturally, not all names are truly ambiguous in all domains and corpora. In these cases a classifier could gain by simply memorizing the names. We made an experiment in which we used only the ambiguous gene and protein names as data points and the performance obtained was over 95%. Using context words as extra features improved the performance only by 0.3% percentage points, with optimal context span 1 found experimentally. The difference was not statistically significant. To assess the performance in a more general setting and to avoid the overfitting effect of memorizing, we do not include the instance of the term to be disambiguated into its context, as the purpose of this paper is to study context-based name disambiguation. Note also that memorizing the names does not help the classifier to disambiguate names that do not exist in training data, for example, names introduced only recently.

The text was further preprocessed by removing stop words and stemming the words with the Porter stemming algorithm [47] (stemming and stop-word removal are discussed in the Methods section).

### Measure of performance

The number of protein examples (30269) in our corpus is about three times greater than the number of gene examples (9533). Thus, we could achieve a classification accuracy of about 75% by always predicting the protein class. To cope with the imbalance in the data, we measure the performance of each classifier as the area under ROC curve (AUC). ROC curve is a relation between the true-positive rate (TPR) and the false-positive rate (FPR) at various classification thresholds:



where *TP*, *FN*, *FP*, and *TN *are true positives, false negatives, false positives, and true negatives, respectively. Unlike other popular measures such as accuracy and precision-recall analysis, the AUC measure is invariant to the prior class probabilities. AUC corresponds to the probability that given a randomly chosen positive example and a randomly chosen negative example, the classifier will correctly say which is which. For a thorough discussion of ROC curves and the AUC measure, see, for example, Fawcett [41], Maloof [48], and Bradley [49].

We cross-validate all AUC measurements using the 5 × 2 cross-validation scheme, which is an ordinary 2-fold cross-validation performed five times. To obtain a 2-fold cross-validated performance estimate, we randomly divide a set of abstracts into two equally-sized sets and average the two performance measurements obtained by training the classifier on one set and testing the classifier on the other set. To obtain a 5 × 2 cross-validated performance estimate, a 2-fold cross-validation is performed five times and the estimates are then averaged. To avoid indirect overlap between test and training sets, we form the sets so that examples originating in the same abstract always remain in the same set.

To test for statistical significance, we use the robust 5 × 2-cv test [50]. The test avoids the problem of dependence between folds in *N*-fold cross-validation schemes and results in a more realistic estimate than, for example, the *t*-test.

### Experimental setup

We randomly divided the preprocessed set of 2000 abstracts into two equal-sized sets; 1000 abstracts for parameter estimation and 1000 abstracts for final validation of the methods. The 5 × 2 cross-validated AUC was used as the measure of performance in both parameter estimation and final validation. In all the experiments with SVMs, we normalized the word frequency vectors to unit length, because the sizes of the contexts varied considerably. We carried out the SVM experiments using the LIBSVM 2.6 software [51] and the Naive Bayes experiments using the Bow toolkit [52].

**Figure 2:**
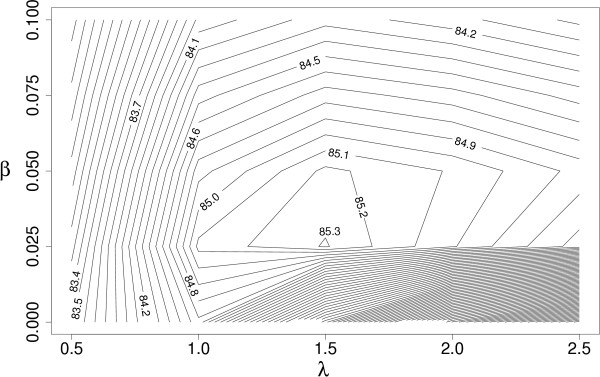
**Parameter estimation: The performance of the weighted SVM with the linear kernel as a function of the weighting parameters *λ *and *β*. **The best performance is reached at *λ *= 1.5 and *β *= 0.025.

**Figure 3:**
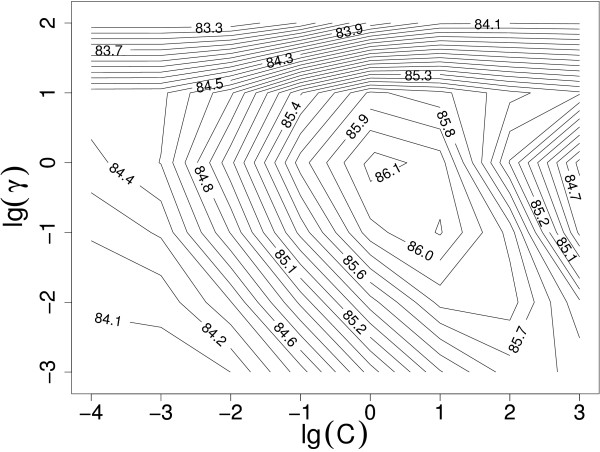
**Parameter estimation: The performance of the weighted SVM with the Gaussian kernel as a function of the SVM penalty parameter *C *and the kernel parameter *γ*. **Both parameters are in a logarithmic scale. The best performance is reached at *C *= 1 and *γ *= 1.

In the experiments, optimal parameter values for SVMs with different kernels and for the proposed weighting scheme must be searched (the exact definitions of the parameters and kernel functions are presented in the Methods section). Every SVM itself has always a penalty parameter *C*, linear kernel has no other parameters than *C*, and both Gaussian and polynomial kernels have an additional parameter *λ*. Adopting a contextual representation yields a context span parameter *s*. The SVM equipped with the weighting scheme has two additional parameters *λ *and *β *by which we may control the effect of the distances of the words from the name to be disambiguated when weighting the context words. The performance of a classifier may be strongly influenced by the choice of the values for its parameters. For example, from Figure 3 (discussed in more detail later) it can be observed that a wrong choice of the kernel parameters as well as the SVM penalty parameter *C *can lead to a severe loss in performance. Particularly when comparing the methods, the correct parameter setting for each of the compared methods is crucial, as only then a reliable estimate of the performance is obtained for each of the methods. The correct parameter values cannot be known in advance and the use of the default values may result in sub-optimal classification performance. Therefore, the parameter values are most commonly estimated from the data. Hsu et al. [45] recommend a grid-search on *C *and *γ *parameters using cross-validation and exponentially growing sequences of *C *and *γ*. Since an exhaustive search for the parameters can not be done in the continuous space of SVM and kernel parameters, we performed a coarse preliminary search in order to find an auspicious region, and subsequently conducted a finer grid search. As can be observed from Figure 2, the choice of values of the weighting parameters *λ *and *β *is as important for the classification performance as the choice of the other parameters. Moreover, as noticed by Ginter et al. [26], the optimal values depend on the task and are not known beforehand. Therefore, we use a grid-search also for finding the optimal values of the parameters *λ *and *β*.

In short, the parameter estimation for SVM classifiers was performed as follows. First we estimated the context span *s *for the conventional SVM and the values of *λ *and *β *for the weighted SVM, using a grid search with the linear kernel function. We used the whole abstract to form the examples for the weighted SVM. With the *s*, *λ *and *β *parameters fixed, we evaluated different types of SVM kernels, estimating the kernel parameters with a grid search. The SVM penalty parameter *C *is optimized separately at each point of the context span, weighting and kernel parameter grids.

In a similar manner, the optimal combination of *λ*, *β *and *s *for the WAC classifier was found by performing a 3-dimensional grid search. For the Naive Bayes classifier, only the optimal value for *s *must be searched. For final validation, we chose the best performing kernel function for conventional and weighted SVMs. Using the parameter values found in the parameter estimation phase, we then measured the performance of each of the compared classifiers on the validation set, again using 5 × 2 cross-validation.

A detailed explanation of the grid search for the parameter estimation described above is presented in the following sections.

### Parameter estimation for conventional SVM

First, we searched for the optimal context span *s *that we will use in our experiments with the conventional SVM. We experimented with different context spans using the conventional SVM with the linear kernel, namely spans of 1, 2, 3, 4, 5, 10, 20, 30, 40, 50, 60, 70, 80, 90 and 100 words to both directions from the term to be disambiguated. The parameter *C *for the SVM with the linear kernel was searched with values 2^-5^, 2^-4^,..., 2^3 ^for each of the different context spans. In these experiments, the context span of 60 words to both directions from the term to be disambiguated resulted in the highest performance for the conventional SVM. We used this context span when experimenting with Gaussian and polynomial (*d *= 2, *d *= 3) kernels, because simultaneous searching for optimal context span and kernel parameters *C *and *γ *for the Gaussian kernel and polynomial kernels with degrees *d *= 2 and *d *= 3 would have been computationally impractical. The values of the *C *and *γ *parameters of the Gaussian kernel were 2^-5^, 2^-4^,..., 2^2 ^and 2^-3^, 2^-2^,..., 2^2^, respectively, and the values for the *C *and *γ *parameters of the polynomial kernels were 2^-10^, 2^-9^,..., 2^-2 ^and 2^-2^, 2^-1^,..., 2^5^, respectively. The results obtained with different kernel functions using the optimal context span *s *= 60 found with the linear kernel are shown in Table 1.

**Table 1:** Parameter estimation: The performance of conventional SVMs with different kernel functions, context span *s *= 60.

Kernel	AUC	Parameters
Linear	80.47%	*C *= 2^-3^
Gaussian	80.62%	*C *= 2^-2^, *γ *= 2
Polynomial (*d *= 2)	80.49%	*C *= 2^-9^, *γ *= 8
Polynomial (*d *= 3)	80.53%	*C *= 2^-7^, *γ *= 2

### Parameter estimation for weighted SVM

With the weighted SVM, we always used the whole abstract as a context. For the weighted SVM, we estimated the best combination of the weighting parameters *λ *and *β *with the linear kernel. The parameter *C *was also separately searched with values 2^-5^, 2^-4^,..., 2^3 ^for each of the different weightings. The comparison of the performance with different weightings using the linear kernel is illustrated in Figure 2. At this point, we found that the values *λ *= 1.5 and *β *= 0.025 performed best for the linear kernel (for illustration, see Figure 6). We used these parameters when experimenting with Gaussian and polynomial (*d *= 2, *d *= 3) kernels. The values of the *C *and *γ *parameters of the Gaussian kernel were 2^-4^, 2^-3^,..., 2^3^and 2^-3^, 2^-2^,..., 2^2^, respectively, and the values for the *C *and *γ *parameters of the polynomial kernels were 2^-7^, 2^-6^,..., 2^-2 ^and 2^-2^, 2^-1^,..., 2^2^, respectively. The performance of the weighted SVM with different *C *and parameters of the Gaussian kernel is illustrated in Figure 3. The figure illustrates the importance of correct parameter selection. The weighted SVM performance with different combinations of *γ *and the penalty parameter *C *follows the behavior described by Keerthi and Lin [53]: Areas of underfitting can be seen at the left, where the value of the *C *parameter is low, and at bottom left where the values of both *C *and *γ *are low. On the other hand, SVM with Gaussian kernel overfits heavily if the value of *γ *is too large, as can be seen at the top of the figure. Overfitting happens also with noisy data at the right part of the figure, where the value of *C *is too large. The results obtained with different kernel functions using the best weighting parameters *λ *= 1.5 and *β *= 0.025 found with the linear kernel are shown in Table 2.

**Table 2:** Parameter estimation: The performance of weighted SVMs with different kernel functions, *λ *= 1.5 and *β *= 0.025.

Kernel	AUC	Parameters
Linear	85.31%	*C *= 1
Gaussian	86.15%	*C *= 1, *γ *= 1
Polynomial (*d *= 2)	86.09%	*C *= 2^-3^, *γ *= 2
Polynomial (*d *= 3)	86.13%	*C *= 2^-3^, *γ *= 1

### Parameter estimation for baseline methods

**Figure 4:**
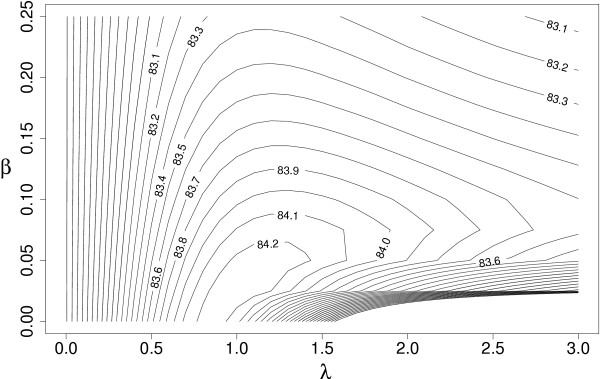
**Parameter estimation: The performance of WAC with *s *= 55 as a function of the weighting parameters *λ *and *β*. **The best performance is reached at *λ *= 1.0 and *β *= 0.025.

The only parameter of the Naive Bayes classifier is the context span *s*. We performed a search for *s *∊ [5, 30] with step 5. The performance reached maximum for *s *= 15. The WAC incorporates an identical weighting scheme as the weighted SVM. We found the optimal parameters by performing a 3-dimensional grid search for *λ *∊ [0,3] with step 0.1, *β *∥ [0, 0.25] with step 0.025 and context span in the interval [5,70] with step 5. The maximum performance was obtained for *λ *= 1, *β *= 0.025 and context span *s *= 55 (for illustration, see Figure 4).

### Final validation

**Figure 5:**
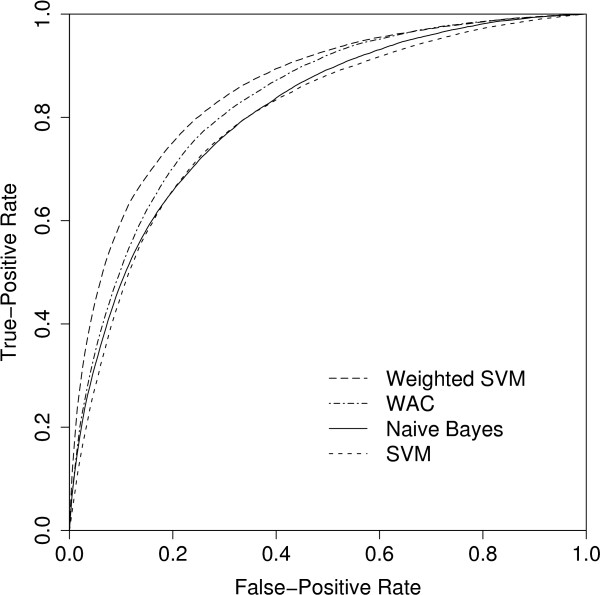
**Final validation: Averaged ROC curves of the classifiers. **The averaged ROC curves were obtained from the folds of the 5 × 2 cross-validation using the vertical averaging method described by Fawcett [41].

The Gaussian kernel was found to be the best with both the conventional and weighted SVMs when tested in the parameter estimation. The best parameters for the Gaussian kernel were *C *= 0.25 and *λ *= 2 with the conventional SVM, and *C *= 1 and *λ *= 1 with the weighted SVM. The AUC results of the final validation are presented in Table 3 and the ROC curves are given in Figure 5. To test the statistical significance of AUC differences between the weighted SVM, the conventional SVM, WAC and Naive Bayes, we performed the robust 5 × 2-cv test on the validation data. Each of the pairwise differences were strongly significant with *p*-values below 0.01, except for the difference between the conventional SVM and the Naive Bayes classifier (*p*-value of about 0.1). The conventional SVMs performed poorly compared to the baselines, especially to the WAC classifier that takes advantage of the contextual weighting. Incorporation of the weighting scheme into SVMs, however, improved their performance by five percentage points.

**Figure 6:**
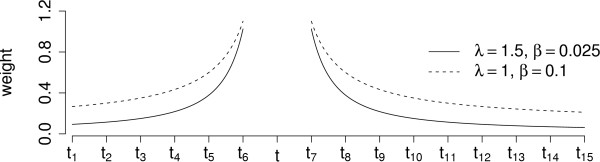
**A weighting example. **Here is an example of a context (*t*_1_,..., *t*_16_). The term *t *is the name to be disambiguated. Figure illustrates the weights of the context words *t*_1_,..., *t*_16 _with two different parameter value pairs of *λ *and *β*. The weight values are represented as a continuous function, although they take discrete values. The parameter combination *λ *= 1.5 and *β *= 0.025 yields the best performance when using the linear kernel.

**Table 3:** Final validation: The performance of the classifiers.

Method	AUC
Conventional SVM	79.85%
Weighted SVM	85.48%
WAC	83.05%
Naive Bayes	80.81%

## Conclusion

In this paper, we show that SVMs can be successfully applied to gene versus protein name disambiguation. We demonstrate how their performance can be further improved by incorporating a weighting scheme based on the intuition that the words near the name to be disambiguated are more important than the other words. The weighting scheme results in a notable performance gain of five percentage points. We also study carefully the effects of different kernel functions and parameters and show that the proposed weighting scheme influences the performance even more than the selection of the kernel part of SVMs. The weighted methods statistically significantly outperformed their unweighted counterparts, the difference being particularly notable for SVMs.

In Ginter et al. [26], we have shown that the optimal values for *λ *and *β *are non-zero and differ substantially depending on the classification task at hand. This suggests that the extent to which the long distance words contribute to the classification is task-dependent and could reflect differing properties of the tasks. While finding correct values of these parameters is clearly important as shown by the experiments, an exact interpretation of the values remains speculative. However, we discuss several reasons why the use of the proposed weighting scheme is beneficial. Further study could bring a better insight into the underlying phenomena.

The performance of the weighted SVM might be further improved, for example, by using collocations in order to capture the local syntax around the term to be disambiguated. However, the proposed weighting scheme uses the local information, and therefore it already captures the information represented by collocations to some extent. In addition, several special text kernels have successfully been applied to text classification as reported by Lodhi et al. [54] and by Cancedda et al. [55]. These kernels and different weighting methods based on the distances and also, for example, on the biological relevance of the words in the context, are still to be studied.

## Methods

In this section, we describe the concepts necessary to understand the application of a SVM classifier to gene versus protein name disambiguation. We start by giving a short introduction to SVMs. The training data points of the classifier are vectors describing the word frequencies in the context in which the names to be disambiguated were found. Then, we explain the general bag of words (BoW) approach that uses only the word frequencies. However, the distances of the words with respect to the word to be disambiguated seem intuitively to be important. We describe a weighting scheme (the weighted BoW) based on distances of the context words from the ambiguous name. Finally, we briefly introduce the two baseline methods, the Naive Bayes classifier and the Weighted Additive Classifier.

### Support Vector Machines

Here, we give a brief description of SVMs. A more comprehensive treatment can be found, for example, in Burges [43] and Vapnik [44]. In a binary classification task *m *labeled examples (*x*_1_, *y*_1_),..., (*x*_*m*_, *y*_*m*_), where *x*_*i *_ε *X *are training data points and *y*_*i *_ε {-1, +1} are the corresponding class labels, form the training set. In order to make the data linearly separable, data points are mapped from the input space *X *to a feature space *F *with a mapping

Φ : *X *→ *F*

before they are used for training or for classification.

SVMs can be considered as a special case of the following regularization problem:



where *i *ranges from 1 to *m*, *l *is the loss function used by the learning machine, *f *: *X *→ *Y *is a function which maps the input vectors *x *∊ *X *to the output labels *y *∊ *Y*, *C *∊ _+ _is a regularization/penalty parameter, and || · ||_*k *_is a norm in a Reproducing Kernel Hilbert Space defined by a positive definite kernelfunction *k*. The second term is called a regularizer. The loss function used by SVMs for binary classification problems is called linear soft margin loss or hinge loss and is defined as

*l*(*f*(*x*), *y*) = max(1 - *yf*(*x*), 0).

By the Representer Theorem, the minimizer of (1) has the following form:



where *a*_*i *_ε  and *k *is the kernel function associated with the Reproducing Kernel Hilbert Space mentioned above.

The penalty parameter *C *controls the trade-o3 between the complexity of the decision function and the number of wrongly classified training points the model will tolerate in the feature space. Minimizing number of training errors by selecting an appropriate parameter can sometimes lead to overfitting due outliers. On the other hand, too strong regularization (low penalization) underfits. A good insight of trade-o3 can be found, for example, in Hastie et al. [56].

There are several commonly used kernels (see Vapnik [44]). The ordinary inner product is called the *linear kernel*

*k*(*u*, *v*) = <*u*, *v>*

and the *polynomial kernel *is defined as

*k*(*u*, *v*) = (*γ*<*u*, *v*> + 1)^*d*^

where *d *ε  is the degree of the polynomial and *γ *ε _+_. When the polynomial kernel is used, the datapoints are mapped into a feature space which contains all products of input vector elements up to *d *(see e.g. Vapnik [57]). The *γ *parameter of the polynomial kernel controls the weight differences of the product features of different orders. Another widely used kernel function is the *Gaussian kernel*



whose width is determined by the *γ *parameter. We refer to Keerthi and Lin [53] for more information of the behavior of the SVM with the Gaussian kernel with different combinations of and the penalty parameter *C*. Hsu et al. [45] suggested to use a cross-validation and a grid search in order to estimate the best combination of and the penalty parameter *C *for the Gaussian kernel. We adopt this procedure and also perform a similar search for the linear and polynomial kernels. A detailed description of the parameter selection is given in the Results and Discussion section.

### Representation of contexts

In our experiments, we trained the SVM classifier to disambiguate the sense of a term between two possible senses based on its context. Let us denote by *s *the *context span *parameter controlling the lengths of the contexts. For a fixed *s*, we take such a context  = (*t*_1_,..., *t*_*l*_) that both the number of words preceding and following *t *is maximal but at most *s*. The words which precede *t *are *t*_1_,..., *t*_*k*_, 0 ≤ *k *≤ *s*, in the order they appear in the text, and correspondingly *t*_*k*+1_,..., *t*_*l*_, 0 ≤ *l *- *k *≤ *s *are the words which follow *t *in the text.

Hence, if there exist *s *words preceding and following the word to be disambiguated, then *k *= *l *- *k *= *s*. The contexts may be of different lengths, since the number of words from *t *to the beginning or end of the abstract may be smaller than *s*.

### The BoW approach

Let *C *be a set of all possible contexts and let *V *= {*v*_1_,..., *v*_*n*_} be an ordered set of all distinct words of the contexts of *C*. We formed the set *V *of all distinct words separately for each training-testing experiment from the words found in the contexts of abstracts of the training set. Let be the mapping, which maps contexts to BoW vectors, defined by



where , 1 ≤ *i *≤ *n*, is the number of occurrences of the word *v*_*i *_∊ *V *in the context . Thus, only the frequency of a word in the context is recorded, but the information about the distances of the words from the ambiguous name is ignored. The BoW vectors can now be used as input space data points for SVM classifiers.

The number of words in *V *is usually large, and therefore the dimension of the input space is also high. Customary means to reduce the dimensionality are, for example, stop-word removal and stemming. Stop-words are words that occur very often in all documents, for instance, 'is', 'the', 'are', 'a'. Stemming combines words that only differ in suffix. For example, the stemming algorithm of Porter [47] removes all suffixes it recognizes. We have applied these dimensionality reduction techniques in our main experiments. However, the number of words still often remains in tens of thousands.

In a separate experiment, we have estimated the effect of stop word removal and stemming using theconventional SVM with the linear kernel and grid search optimization of *C *and context span parameters. We found that stemming results in a low increase in performance (0.59%), stop-words removal haspractically no effect on the performance (an increase of 0.01%). Neither of the differences were statistically significant.

### The weighted BoW approach

The BoW approach does not preserve any information about the positions of the words in the context. Therefore, we use a particular weighting scheme based on the distances of the words from the term *t *to be disambiguated. The idea is that the words near *t *are more likely to be important than other words, and therefore they are given larger weights.

The weighted vector space model of contexts can be formalized as follows. Let dist(*j*) denote the distance of the word *t*_*j *_from the term *t*, that is, the number of words between the word *t*_*j *_and *t *including the word *t*_*j *_itself. Let further Pos(*v*, ) = {*j *| *v *= *t*_*j *_ε } denote a set of positions *j *for each word *v *ε *V *in a particular context . The weight for the word *t*_*j *_is defined as



where *λ*, *β *≥ 0 are the parameters for the weighting. If *λ > *0, the weights get a hyperbolic shape with highest values immediately around the term to be disambiguated (see Figure 6). The bigger *λ* is, the steeper the weight values grow towards the term *t*, and *β *is an offset of the values. The role of *β *is to reduce the ratio between the weights of the words that are near to the term *t *and the weights which are far from *t*.

Let Ψ now be the function which maps contexts to weighted BoWs given by



Where



Note that the setting *λ *= *β *= 0 corresponds to the ordinary BoW approach (2).

### Baseline methods

The two baselines, the Naive Bayes classifier and the Weighted Additive Classifier, represent a family of linear classifiers based on aggregating the class-wise co-occurrence statistics of the words in the context. The Naive Bayes classifier (see, for example, Manning and Schütze [23]) evaluates the a posteriori conditional probability of a class by computing a product of the corresponding conditional probabilities of the context words obtained from their class-wise co-occurrence statistics.

The Weighted Additive Classifier [26] considers co-occurrence statistics similar to that used in the Naive Bayes classifier. However, the decision rule is additive and incorporates a weighting scheme. The weighting can be defined in a manner identical to that described in the section on weighted BoW.

## Authors' contributions

TP carried out the experiments with SVMs and the weighting scheme as well as the sense distribution analyses. He also drafted the manuscript. FG designed and carried out all the experiments with Naive Bayes and WAC. JB, JJ and TS conceived the original design of the work, participated in the analysis and interpretation of data, and provided scientific guidance. All authors critically revised the manuscript and approved the final version.
